# Effects of Adenotonsillectomy on Serum Levels of IGF-1 and IGFBP-3 and Growth Indices in Children with Adenotonsillar Hypertrophy or Recurrent Tonsillitis

**Published:** 2016-09

**Authors:** Mohammad Farmarzi, Mahmood Shishegar, Seyed Taghi Heydari, Arash Haghighi, Hadi Sharouny

**Affiliations:** 1 *Department of Otorhinolaryngology Head and Neck Surgery, Khalili Hospital, School of Medicine, Shiraz University of Medical Sciences, Shiraz, Iran.*; 2 *Health Policy Research Center, Shiraz University of Medical Sciences, Shiraz, Iran.*; 3 *Department of Otorhinolaryngology Head and Neck Surgery, Baqiyatallah Hospital, School of Medicine, Baqiyatallah University of Medical Sciences, Tehran, Iran.*

**Keywords:** Adenoidectomy, Adenoids, Tonsillectomy, Insulin-Like Growth Factor I, Insulin-Like Growth Factor-Binding Protein 3, Palatine tonsil

## Abstract

**Introduction:**

Adenotonsillar hypertrophy (ATH) may present with growth retardation. Insulin-like growth factor 1 (IGF-1) mediates the anabolic effects of growth hormone (GH) on tissues. Most of the circulating IGF-1 molecules are bound to insulin-like growth factor-binding protein 3 (IGFBP-3). IGF-1 and IGFBP-3 serum levels reflect the levels of daily mean serum GH and are used as indices for evaluating the serum level of GH. This study aimed to determine the effect of adenotonsillectomy on IGF-1 and IGFBP-3 serum levels in patients with ATH or recurrent tonsillitis. Furthermore, we aimed to investigate the effect of adenotonsillectomy on growth indices such as weight and height.

**Materials and Methods:**

A total of 100 randomly selected children with a diagnosis of ATH or recurrent tonsillitis with a mean age of 10.2 ± 1.4 years (range, 3-17 years) were enrolled in the intervention group. Of those, 53 were boys and 47 were girls. The control group included 100 healthy children (62 boys and 38 girls) with a mean age of 8.5 ± 1.5 years (range, 4-15 years). Growth indices such as weight and height were measured and documented at the time of surgery and 6 months after the operation. Blood samples were taken preoperatively and repeated 6 months after adenotonsillectomy. The coated-tube immunoradiometric (IRMA) method was used to measure IGF-1 and IGFBP-3 levels.

**Results:**

Postoperative IGF-1 and IGFBP-3 serum levels as well as weight and height showed were significantly greater in comparison with preoperative measurements in both the intervention and control groups (P<0.001). At the end of study, the intervention group showed significantly greater changes from baseline in IGF-1 and IGFBP-3 serum levels, weight, and height in comparison with the control group (P< 0.001).

**Conclusions:**

This study shows that adenotonsillectomy in children with ATH or recurrent tonsillitis increases IGF-1 and IGFBP-3 serum levels in comparison with preoperative levels by affecting the GH-IGF-1 axis, and subsequently leads to a faster increase in growth indices compared with healthy peers during the same period.

## Introduction

Adenotonsillar hypertrophy (ATH) is the apparent cause of recurrent tonsillitis and upper airway obstruction in pediatric patients. A reduced dietary intake and failure to gain sufficient weight is often reported by parents of children with a history of recurrent acute tonsillitis (1,2). 

The pathophysiology of failure to thrive (FTT) due to ATH includes low calorie intake, increased energy requirements associated with labored breathing, hypoxemia, and interruption of growth hormone–insulin-like growth factor-1 (GH-IGF-1) axis secretion (3,4).

Anabolic and growth-inducing effects of GH are primarily mediated by inducing the expression of IGF-1 in the liver and peripheral tissues (5-7). Virtually all of the circulating IGF-1 molecules are bound to a carrier protein called insulin-like growth factor-binding protein 3 (IGFBP-3). IGF-1 and IGFBP-3 serum levels reflect the levels of daily mean serum GH and are used as indices for evaluating the serum level of GH.

Adenotonsillectomy is considered the treatment of choice in patients with ATH and recurrent tonsillitis. Absolute indications for adenotonsillectomy include ATH with obstructive sleep apnea (OSA), FTT, aberrant dentofacial growth, or suspicion of malignancy. Relative indications include ATH with blockage of upper airways, dysphagia, speech impairment, or halitosis (8,9).A limited number of high-quality studies have been conducted into the effect of adenotonsillectomy on growth indices thus far. The aim of this study was to investigate the effect of adenotonsillectomy on the IGF-1 and IGFBP-3 serum levels and growth indices such as height and weight in children with ATH.

## Materials and Methods

*Study population*

Patients of children aged 1–18 years old who would most benefit from tonsillectomy were identified according to the latest evidence-based guidelines of the American Academy of Otolaryngology–Head and Neck Surgery (AAO–HNS) (9). A diagnosis of recurrent tonsillitis was based on hospital documentation and a detailed physical examination. Children with any underlying disease predisposing to upper airway obstruction, asthma, allergic rhinitis, or chronic disease were excluded from the study. After obtaining informed consent from the guardians of the children, 53 boys and 47 girls were enrolled in the study as an intervention group, with a mean age of 10.2 ± 1.4 years (range, 3–17 years). The control group included 100 children (62 boys and 38 girls) with a mean age of 8.5 ± 1.5 years (range, 4–15 years).

Puberty was defined as an age range of 14–17 years in boys and 8–13 years in girls (10). The intervention and control groups were both divided into prepubertal and pubertal subgroups. Study subjects were randomly selected from patients who were referred to the outpatient Clinic of Otorhinolaryngology – Head and Neck Surgery, Shahid Ayatollah Datgheib Hospital, Shiraz, IR Iran, from June to December 2008. Patients were referred because of recurrent tonsillitis or obstructive sleep disorders due to ATH. Children in the control group were selected from healthy children who presented to the primary healthcare center of the same hospital. 

Adenoid hypertrophy was defined as obstruction of more than 50% of the nasolaryngeal space, as confirmed by preoperative imaging or intraoperative evaluation. Adenotonsillectomy was performed by a cold dissection curettage technique, and bipolar cautery was used for hemostasis.

*Data Collection*

Measurement of growth indices

Preoperative and postoperative evaluation including a detailed physical examination, measurement of height and weight, and sampling for routine blood tests were performed. A trained nurse in the outpatient clinic measured all preoperative heights and weights. The same scale was used for all preoperative and postoperative weight and height measurements, and the accuracy of the scale was verified. Weight was rounded to the nearest 0.1 kg using a balance beam scale while height was rounded to the nearest 1.0 mm with wall-mounted stadiometer during the preoperative period and 6 months after surgery.

*Blood Sampling*

The first venous blood sample was drawn before surgery and the second was obtained 6 months after the procedure. In addition, blood sampling from the control group was performed twice within a 6-month period.

*Laboratory Methods*

All blood samples taken preoperatively and postoperatively were centrifuged immediately and stored at −20 °C until the assays were performed. Serum was studied by the coated-tube immunoradiometric assay (IRMA) method using a commercial kit (Diagnostics Systems Laboratories Inc, Webster Texas, USA). All procedures were performed by the same technician in the laboratory. The accurate determination of all serum levels of IGF-1 and IGFBP-3 was performed in one assay.

*Statistical analysis*

Statistical analysis was performed using SPSS 15 (SPSS Inc, Chicago, Illinois, USA). Measurement differences were calculated by subtracting the preoperative data from the postoperative data. The paired t-test was used to assess the changes in the serum levels of IGF-1, IGFBP-3, and growth indices from baseline to the end of the study in each group. The differences between preoperative and postoperative data were calculated and compared between the intervention and control groups by independent samples t-test. A two-tailed P<0.05 was considered statistically significant.

## Results

In comparison with the baseline values, the postoperative IGF-1 and IGFBP-3 serum levels, weight, and height were significantly increased in both groups (P<0.001) (Table.1).

**Table 1:** Comparison of preoperative and postoperative IGF-1 and IGFBP-3 serum levels, weight, and height

		**Baseline, Mean ± SD**	**Postoperation, Mean ± SD**	**P-value**
IGF-1, ng/ml	Case	473.4 ± 114.9	557.4 ± 110.2	<0.001
	Control	489.5 ± 114.4	522.2 ± 124.4	<0.001
IGFBP-3, ng/ml	Case	3256.4 ± 118.1	3375.6 ± 148.2	<0.001
	Control	3291.4 ± 118.8	3352.2 ± 120.2	<0.001
Weight, kg	Case	31.6 ± 9.1	35.5 ± 8.9	<0.001
	Control	33.5 ± 9.1	34.9 ± 9.1	<0.001
Height, cm	Case	103.2 ± 18.1	106.3 ± 18.0	<0.001
	Control	104.3 ± 18.1	105.4 ± 18.2	<0.001

IGF-1, insulin-like growth factor 1; IGFB-3, insulin-like growth factor-binding protein 3

Comparison of the differences in IGF-1 and IGFBP-3 serum levels, weight, and height showed a statistically significant difference between intervention and control groups (P< 0.001) (Table.2).

**Table 2:** Comparison of differences in IGF-1 and IGFBP-3 serum levels, weight, and height between intervention and control groups

		**Changes from Baseline, Mean ± SD**	**P-value**
IGF-1, ng/ml	Case	84.0 ± 44.4	<0.001
	Control	32.7 ± 36.5	
IGFBP-3, ng/ml	Case	119.2 ± 89.1	<0.001
	Control	60.7 ± 68.5	
Weight, kg	Case	3.8 ± 1.5	<0.001
	Control	1.3 ± 1.0	
Height, cm	Case	3.1 ± 1.1	<0.001
	Control	1.1 ± 0.5	

IGF-1, insulin-like growth factor 1; IGFB-3, insulin-like growth factor-binding protein 3

Differences in IGF-1 and IGFBP-3 serum levels, height and weight were not significantly different in prepubertal and pubertal participants between the intervention and control groups (Table.3).

**Table 3:** Comparison of differences in IGF-1 and IGFBP-3 serum levels, weight, and height between prepubertal and pubertal groups

		**Puberty**	**No.**	**Changes from baseline, Mean ± SD**	**P-Value**
**IGF-1, ng/ml**	Case	Prepubertal	45	88.3 ± 44.6	0.388
		Pubertal	55	80.5 ± 44.4	
	Control	Prepubertal	45	32.1 ± 37.4	0.887
		Pubertal	55	33.1 ± 36.1	
**IGFBP-3, ng/ml**	Case	Prepubertal	45	118.8 ± 78.3	0.971
		Pubertal	55	119.5 ± 97.8	
	Control	Prepubertal	45	64.7 ± 69.7	0.603
		Pubertal	55	57.5 ± 68.0	
**Weight, kg**	Case	Prepubertal	45	4.1 ± 1.4	0.189
		Pubertal	55	3.7 ± 1.6	
	Control	Prepubertal	45	1.5 ± 1.4	0.138
		Pubertal	55	1.2 ± 0.6	
**Height, cm**	Case	Prepubertal	45	3.2 ± 1.5	0.317
		Pubertal	55	3.0 ± 0.6	
	Control	Prepubertal	45	1.0 ± 0.5	0.196
		Pubertal	55	1.1 ± 0.5	

IGF-1, insulin-like growth factor 1; IGFB-3, insulin-like growth factor-binding protein 3.

## Discussion

The prevalence of ATH-associated growth retardation has been reported previously as 1–46% in various studies (5,11-14). Although a reduced dietary intake and failure to gain weight is generally seen in pediatric patients with ATH, its exact mechanism remains unclear. Growth improvement after surgical removal of the nasopharyngeal airway blockage is noticeable, probably due to multiple factors such as increased GH secretion, increased energy intake, and decreased energy consumption (13–15).

GF-1 and IGFBP-3 serum levels reflect the levels of daily mean endogenous serum GH. The excellent reproducibility of IGF-1 and IGFBP-3 on regular testing makes them ideal parameters for the assessment of the GH-IGF-1 axis. Measuring the serum levels of IGFBP-3 may be an alternative to GH profiles in many cases, where GH secretion rate evaluation is required (16). In healthy children, IGF-1 and IGFBP-3 serum levels indicate slight diurnal fluctuation. Measurement of exact IGF-1 and IGFBP-3 serum levels has been widely used as a screening parameter in the assessment of growth disorders. The serum level of IGF-1 is greatly affected by chronologic age, nutritional status, and degree of sexual maturation. 

IGF molecules bind to a family of proteins named IGFBP and form a complex in the plasma. IGFBP-3 is the main IGFBP in the normal human serum and shows a clear dependence on GH levels. It is proposed that measurement of serum IGFBP-3 by IRMA might be better than IGF-1 assays in the diagnosis of GH deficiency, because young children may have very low levels of IGF-1. Moreover, IGFBP-3 estimations represent not only IGF-1 level but also IGF-2 level, and its age dependency is not as noticeable as that of IGF-1. In addition, IGFBP-3 measurement by IRMA is technically simple and the level of IGFBP-3 in normal serum is quite high; thus this assay has a high sensitivity (17).

In a study by Chiba et al., eight out of ten patients showed an increase in IGF-1 after tonsillectomy, while an increase in urinary GH levels was seen in seven patients (18). This study is the only one found in the literature that demonstrated an increase in GH levels after tonsillectomy. 

Nevertheless, the determination of IGF-1 concentration in serum is more reliable than urinary GH levels, because serum levels of IGF-1 reflect mean daily serum levels of GH.

Several previous studies have evaluated the effects of adenotonsillectomy on IGF-1 and IGFBP-3 serum levels and growth indices in children with ATH (Table.4).

**Table 4:** Summary of previous studies evaluating the effects of adenotonsillectomy on IGF-1 and IGFBP-3 serum levels and growth indices in children with adenotonsillar hypertrophy.

**Study (Year) **	**No.**	**Age Range (y)**	**Follow-up Period (months)**	**Control Group**	**Lab Method**	**Result**
Bar et al^(3)^ (1999)	13	6 ± 2.8	3–12	No	ELISA	IGF-1↑ IGFBP-3↔ Weight↑ Height↑
Dualibi et al^(19) ^(2002)	7	2–10	4	Yes	Not mentioned	Weight↑ Height↑
Nieminen et al^(20)^ (2002)	70	2.4–10.5	6	Yes	RIA	BMI↑
Yilmaz et al^(5)^ (2002)	41	2–8	3–6	No	RIA	IGF-1↑ IGFBP-3↑
Mitchell et al ^(21)^ (2004)	30	3–17	8.5	No	Not mentioned	BMI↔ statistically not significant
Ersoy et al^(22) ^(2005)	28	3–10	1 y	Yes	ELISA	IGF-1↑ IGFBP-3↑ Weight↑ Height↑
Aydogan et al^(23)^ (2005)	38	4–10	12–18	No	RIA	IGF-1↔ IGFBP-3↔
Vontetsianos et al^(16)^ (2005)	57	2–8	6–13	No	RIA	IGF-1↔ GH↔
Majidi et al^(24)^ (2007)	23	3–10	3 weeks	No	RIA	IGF-1↑ IGFBP-3↑
Fernandes et al^(25)^ (2008)	22	3–9	6	Yes	Not mentioned	Weight↑ Height↑
Kang et al^(26)^ (2008)	52	2.9–10.8	5 years	No	RIA	IGF-1↑ Weight↑ Height↑ BMI↑
Gümüssoy et al(27) (2009)	40	2–10	6	No	ELISA	IGF-1↑ IGFBP-3↑
Kiris et al(28) (2010)	96		6	No	Not mentioned	IGF-1↑ IGFBP-3↑ Weight↑ Height↑
Jabbari Moghaddam et al (29) (2013)	40	6.5±1.2	12 mo	No	ELISA	IGF-1↑ Weight↑ Height↑ BMI↑

ELISA, enzyme-linked immunosorbent assay; RIA, Radioimmunoassay; GH, growth hormone; IGF-1, insulin-like growth factor 1; IGFB-3, insulin-like growth factor-binding protein 3; BMI, body mass index.

In current study, the IGF-1 and IGFBP-3 serum levels were measured 6 months after adenotonsillectomy to avoid any effect of “surgery” on these levels.

In present study, significantly lower serum levels of IGF-1 in the intervention group were seen at the preoperative period when compared with the control group. The increase in serum IGFBP-3 levels was statistically significant. However, the increase in serum IGFBP-3 levels was lower than the increase in serum IGF-1 levels. This can be explained by the binding of IGFBP-3 to both IGF-1 and IGF-2. The blood levels of IGF-2 are not completely determined by GH (18). Moreover, the correlation between IGF-1 and IGFBP-3 serum levels is exponential. Although molecules are regulated by the same factors, the magnitude of variation in the serum levels of IGF-1 is always much greater than that of IGFBP-3. This means that IGF-1 responds more markedly to regulators than IGFBP-3 does (15).

Weight is considered an important parameter in the assessment of infants’ well-being and in any phase of life where nutrition has an important effect on the growth process. In our study, patient weight increased more significantly 6 months after the procedure operation in the intervention group than in the control group. A greater increase in height was detected in surgically treated children during the 6-month period postoperatively, when compared to that of their healthy peers. Weight and height differences in all ages in children with ATH showed a significant increase after adenotonsillectomy in comparison with the control group.

The majority of previous studies showed a significant increase in plasma levels of IGF-1 and IGFBP-3, weight, height, and BMI in patients with ATH after adenotonsillectomy, with the same result the present study. Few studies indicated that there was no significant change in BMI or IGF-1 and IGFBP-3 serum levels after adenotonsillectomy (Table.4).

There was a highly significant increase in plasma levels of IGF-1 and IGFBP-3, weight, and height in the control and intervention groups in the present study. Comparison of differences of growth indices clearly demonstrated the effect of adenotonsillectomy in children with ATH.

In a meta-analysis performed in 2009, the authors concluded that standardized height, weight, and IGF-1 and IGFBP-3 serum levels were significantly increased following adenotonsillectomy. However, due to the unreliable quality of studies in the meta-analysis, the presence of statistical heterogeneity, and the limited number of publications available for meta-analysis, the authors’ conclusions are not very firm and more studies are required to achieve a satisfactory result (30).

The current study has the largest sample size among studies performed in this area. Another advantage of the present study is that it includes a direct comparison of patient groups with healthy peers. We suggest a new meta-analysis to determine the final result.

## Conclusion

Changes in weight, and height of children with ATH after adenotonsillectomy were significantly higher in comparison to the control group. After 6 months, the children’s growth rate significantly increased, as reflected by growth indices. These changes were accompanied by a significant increase in IGF-1 and IGFBP-3 serum levels. These findings suggest that adenotonsillectomy may influence the GH-IGF-1 axis by increasing serum IGF-1 level, leading to a faster growth rate in comparison with healthy peers. We conclude that adenotonsillectomy can help children with ATH to achieve their optimal growth.
